# Hematological and Serum Biochemical Profiles of the Southern Red Muntjac (*Muntiacus muntjak*) Raised in a Semicaptive Environment in Thailand

**DOI:** 10.1155/vmi/6635279

**Published:** 2025-10-29

**Authors:** Marnoch Yindee, Wallaya Manatchaiworakul, Worada Thammasangwan, Punyisa Saetae, Chayanee Sodthianan, Supak Udompornprasith, Supaporn Teerawattananon, Wilasinee Kanchana, Patcharapol Khumngoen, Worawidh Wajjwalku, Tuempong Wongtawan

**Affiliations:** ^1^Akkhraratchakumari Veterinary College, Walailak University, 222 Thaiburi, Thasala District, Nakhon Si Thammarat 80161, Thailand; ^2^One Health Research Center, Walailak University, 222 Thaiburi, Thasala District, Nakhon Si Thammarat 80161, Thailand; ^3^Veterinary Diagnostic Center, Clemson Livestock Poultry Health, Clemson University, 500 Clemson Rd, Columbia 29229, South Carolina, USA

**Keywords:** barking deer, blood profile, Thailand, wildlife

## Abstract

The southern red muntjac (*Muntiacus muntjak*) is a common species found throughout Southeast Asia. It plays a vital ecological role as a prey species for large wild carnivores, contributing to the maintenance of biodiversity. In addition, this species is farmed for tourism purposes and as an alternative source of meat. However, the lack of data on hematological and serum biochemical parameters has made it challenging to assess the health status of this species and to monitor environmental toxicity. The objective of this study was to investigate the hematological and serum biochemical profiles of southern red muntjacs (*M. muntjak*) and to compare any differences between males and females. A total of 82 individuals were captured, and blood samples from 59 were analyzed using automated hematology and biochemistry analyzers. The results revealed no significant differences between males and females in almost all parameters. When compared with previous studies, several hematological and biochemical parameters in *M. muntjak* differed from those of related species (*M. vaginalis*) and may be due to different capture methods and altitude. These findings provide valuable baseline data for health screening and environmental toxicity assessment in both captive and free-ranging populations of this species.

## 1. Introduction

The red muntjac, also known as barking deer, is the most common deer species in Southeast Asia [1, 2]. It can be divided into two subspecies: the northern red muntjac (*Muntiacus vaginalis*) and the southern red muntjac (*M. muntjak*), with their distribution boundary located at the Isthmus of Kra in Thailand [3]. The southern red muntjacs appear mainly in Brunei Darussalam, Indonesia, Malaysia, and southern Thailand but have become extinct in Singapore [4]. Although it is listed as a species of Least Concern on the IUCN Red List [4], its population has been steadily declining, particularly in Thailand, where it is now classified as near threatened and requires conservation measures [5]. While precise population data for wild deer and muntjacs are lacking, recent observations by forest officers indicate that their numbers have decreased by more than 50% compared to previous estimates [6].

Red muntjacs play an important role in animal species diversity and conservation since they are a major prey species for large wild carnivores such as tigers and leopards [7, 8]. Many conservation attempts have been used for deer conservation in Thailand and other Asian countries, including captive and semicaptive breeding in the zoo or commercial farm in order to repopulate to the forest in the future and be an alternative meat source [9, 10].

One essential aspect of managing the health of captive or semicaptive wild animals is monitoring their well-being and treating any illnesses [11]. Blood testing is a commonly used method for assessing health, detecting diseases, and evaluating welfare in captive wildlife [12, 13]. Moreover, blood analysis also has great potential in studies of ecology and ecotoxicology in free-ranging species [12]. However, the major caveat for field researchers is that the “rules” for human or domestic animal hematology do not always apply to wildlife; information on blood health profiles is still lacking for wildlife, including the red muntjac. The objective of this study was to examine the hematological and serum biochemical profiles of southern red muntjacs (*M. muntjak*) and to compare the differences between males and females.

## 2. Materials and Methods

### 2.1. Animals and Ethics

This study was approved by the Institutional Animal Care and Use Committee of Walailak University (ID 66027). The specimens used in this investigation were obtained from barking deer residing in a semicaptive environment on a privately owned commercial farm in Songkhla province, the southern region of Thailand (GPS location: 6°56′26.0″N, 100°15′25.3″E). The project was conducted between January and February 2025.

A total of 82 barking deer were immobilized for health management and subcutaneous microchip implantation. The immobilization procedure involved the administration of anesthetic agents. A 3-mL gas-pressurized dart, containing a combination of tiletamine–zolazepam (2.0 mg/kg, Zoletil Virbac, Carros, France) and xylazine (1.0 mg/kg, Unovet, Bangkok, Thailand), was delivered via a dart syringe propelled by a blowpipe into the hindquarter muscles [14].

During immobilization, physical examinations and body weight assessments were performed. Animals that exhibited no injuries and no clinical abnormalities were classified as healthy. Individual animals with a body weight greater than 20 kg were classified as adults.

### 2.2. Blood Samples

After anesthetizing the animals, they were positioned in dorsal recumbency, and blood samples were carefully collected from the jugular vein using 18 G 1 ½ disposable needles and 10-mL disposable syringes. The interval between anesthetic administration and blood collection ranged from 5 to 10 min.

Two milliliters of blood was transferred into a 4-mL blood collection tube containing EDTA (BD, Plymouth, UK) as an anticoagulant for hematological analysis. An additional 6 mL of blood was placed into 10-mL tubes without anticoagulants (BD, Plymouth, UK) and allowed to clot for at least 30 min in an upright position within a cooler box to later collect the serum. Samples were transported under refrigerated conditions and processed within 4 h of collection.

### 2.3. Hematology

It is important to note that neither the analyzers nor the selected setting had been specifically validated for use with barking deer blood. To ensure the data reliability of the analyzer data, internal quality control procedures were conducted prior to the experiment using control materials provided by the manufacturers.

Hematological analysis was carried out using a BC-5000 Vet Auto Hematology Analyzer (Mindray Animal Care, Shenzhen, China), employing the caprine setting.

The parameters measured included total white blood cell count (WBC), neutrophil count, lymphocyte count, eosinophil count, monocyte count, basophil count, hematocrit (HCT), red blood cell count (RBC), hemoglobin (HB) concentration, mean cell volume (MCV), MC HB (MCH), MC HB concentration (MCHC), red cell distribution width (RDW), platelet count (PLT), and plasma protein levels.

### 2.4. Serum Biochemistry

Clotted blood samples were centrifuged in the laboratory at 2000 g for 10 min. Serum biochemistry analysis was performed according to the manufacturer's instructions using an SMT-120VP analyzer (Seamaty, Chengdu, China). The analysis included a range of measurands: total protein (TP), albumin (ALB), globulin (GLO), ALB/GLO ratio (A/G), total bilirubin (TB), alkaline phosphatase (ALP), aspartate aminotransferase (AST), creatinine (CREA), blood urea nitrogen (BUN), glucose (GLU), amylase (AMY), sodium (Na^+^), and potassium (K^+^).

### 2.5. Data Analysis and Statistical Analysis

Data and statistical analyses were conducted using Jamovi Version 2.6.19 [15]. The Shapiro–Wilk test was applied to assess the normality of the data. Descriptive statistics included the mean, median, standard deviation (SD), standard error of the mean (SE), and 95% confidence interval, as well as minimum (Min) and maximum (Max) values. Differences between male and female deer were evaluated using the Mann–Whitney *U* test. A *p* value of less than 0.05 was considered statistically significant.

## 3. Results

Samples were selected from semicaptive barking deer, excluding those with overt clinical abnormalities, and were collected. Of 82 deer, 59 samples from apparently healthy adult animals were included for analysis. These samples comprised 28 females and 31 males. The average weight was 23.37 kg, the SD was 3.05, and the median was 23.

### 3.1. Normality of Data

Data normality of hematology and serum biochemistry is shown in Table 1. Using the Shapiro–Wilk test, most parameters showed a *p* value less than 0.05, suggesting a violation of the assumption of normality. From 20 parameters of hematology data, 14 parameters (70.00%) showed a *p* value less than 0.05, while of 14 parameters of serum biochemistry, 11 (78.57%) parameters showed a *p* value less than 0.05.

### 3.2. Hematology and Serum Biochemistry

The differences in hematological and serum biochemical parameters between male and female muntjacs are presented in Tables 2 and 3. The overall hematological profiles, including the mean, median, and reference intervals (95% confidence interval), are summarized in Tables 4 and 5.

Male deer exhibited slightly higher values in certain hematological parameters, such as neutrophil count, monocyte count, and HCT, whereas females had slightly higher levels of eosinophil count, basophil count, RDW, and platelet count. However, when assessed using the nonparametric Mann–Whitney *U* test, no statistically significant differences were observed for most parameters, except for basophil count, which was significantly higher in females (*p*=0.02) (Table 2).

Regarding serum biochemistry, females showed marginally higher levels of ALB, TP, GLO, CREA, glucose, and potassium, while males had higher levels of TB, alanine aminotransferase, ALP, AMY, BUN, and sodium. Nevertheless, none of these differences were statistically significant (*p* ≥ 0.05) (Table 3).

## 4. Discussion

This study is the first in nearly 2 decades to report the hematological and serum biochemical profiles of the southern red muntjac (*M. muntjak*). Previously, a closely related species, the northern red muntjac (*M. vaginalis*), was examined under semicaptive conditions at Nainital Zoo, Uttaranchal, India (GPS: 29°22′50.7″N, 79°28′07.1″E), with animals captured using physical restraint [16]. In addition, there have been reports on the hematology and/or serum biochemistry of other deer species in Thailand. These include the captive rusa deer (*Rusa timorensis russa*, formerly *Cervus timorensis russa*) at Kasetsart University, Kampangsan campus, central Thailand (GPS: 14°01′17.4″N, 99°58′33.1″E), studied under physical restraint [17]; the Eld's brow-antlered deer (*Rucervus eldii thamin*, formerly *C. eldi thamin*) reared semicaptively at Khao Khiew Open Zoo, Chonburi, eastern Thailand (GPS: 13°12′53.3″N, 101°03′25.2″E), immobilized using anesthetic drugs [18]; and the sambar deer (*R. unicolor equinus*, formerly *C. unicolor equinus*) at Nong Kwang Animal Research and Breeding Center, Ratchaburi, western Thailand (GPS: 13°43′13.0″N, 99°38′44.6″E), studied under physical restraint [19]. Comparative hematological and serum biochemical data among these species are presented in Tables 6 and 7, respectively.

Notably, the hematological profiles differed between the two closely related species, with *M. vaginalis* exhibited approximately twice in RBC count (21 × 10^6^ cells/mm^3^) and HCT (52%) observed in *M. muntjak*, other deer species, and goat (Table 6) [21]. In wildlife management, physical restraint is known to induce more acute physiological stress and a higher risk of dehydration primarily due to the activation of the fight-or-flight response compared to chemical immobilization (anesthesia) [22, 23]. The elevated RBC concentration reported by Gupta et al. [16] may therefore reflect acute stress and dehydration associated with physical restraint. This response is mediated by the activation of the sympathetic nervous system, which stimulates the release of catecholamines (epinephrine and norepinephrine) from the adrenal glands, leading to splenic contraction and the subsequent release of erythrocytes into circulation [24, 25]. Similarly, a study in red deer (*C. elaphus*) and white-tailed deer (*Odocoileus virginianus*) reported increased RBC and HCT values under physical restraint compared with chemical restraint [26–28].

However, when comparing the RBC counts between *M. muntjak* (under anesthesia) and *Rusa* spp. (physically restrained) in Thailand, the values were comparable. Another possible explanation for the elevated RBC concentrations reported by Gupta et al. [16] is the influence of high altitude. At high altitude, the reduced partial pressure of oxygen results in lower arterial oxygen saturation (hypoxemia), which stimulates the release of erythropoietin to promote RBC production in the bone marrow, as a compensatory mechanism to enhance the oxygen-carrying capacity of the blood [29].

Regarding serum biochemistry, the A/G ratio in deer species from Thailand, including *M. muntjak* and *R. unicolor equinus*, appears to be lower (value < 1) than that of *M. vaginalis* reported in India [16], which showed values greater than 1 (Table 7). An A/G ratio below 1 indicates a higher concentration of GLO relative to ALB. It has been known that ALB levels can reflect nutritional quality, as a diet low in protein may lead to reduced blood protein levels [30]. Additionally, higher ALB levels have also been reported in red deer subjected to physical restraint compared with those under chemical restraint [28].

GLO is typically linked to the body's inflammatory response to pathogen exposure [31]. In Thailand, the GLO of *M. muntjak* in this study and *R. unicolor equinus* [19] was higher than *M. vaginalis* in India [16]. The difference in the GLO concentration may also be influenced by geographical and climatic factors between the two study sites. *M. vaginalis* in the study by Gupta et al. [16] was sampled at Nainital Zoo, Uttaranchal, India, located in a temperate Himalayan foothill region with a cooler climate and lower pathogen diversity. In contrast, *M. muntjak* in the present study was sampled in Songkhla province, southern Thailand, a tropical environment characterized by high temperature, humidity, and year-round exposure to a wide variety of parasites and microorganisms. These environmental differences may contribute to heightened immune stimulation and elevated GLO levels in Thai *M. muntjak* compared with *M. vaginalis* in northern India.

Furthermore, *M. vaginalis* [16] demonstrated a higher level of BUN compared to *M. muntjak* (Table 7). Higher levels of BUN could suggest high-protein diet [32, 33] or dehydration. Dehydration may be related to physical restraint [28], and potentially due to high altitudes, animals lose more water due to the increased respiration rate (hyperventilation water loss through breathing) and increase urine volume (altitude diuresis) [34].

In north India, Nepal, and Pakistan, *M. vaginalis* has been reported to browse or graze on plant species with relatively high crude protein content, including members of the Fabaceae family (e.g., *Acacia, Albizia, Leucaena, Desmodium, and Pueraria* spp.), as well as certain forbs and young shoots rich in nitrogen compounds [35, 36]. Conversely, in southern Thailand, *M. muntjak* primarily forages in moist evergreen forests dominated by low-protein understory vegetation, bamboo, and fruits, which provide a lower dietary nitrogen load [37].

One limitation of this study was the absence of morphological evaluation of red and white blood cells. Previous research on the closely related species *M. vaginalis* described erythrocytes as having a crescent-like or sickle-shaped morphology [16]. Similar sickle-shaped erythrocytes have been reported in several deer species, including barasingha (*Ru. duvaucelii*), chital (*Axis axis*), fallow deer (*Dama dama*), hog deer (*Axis porcinus*), mule deer (*O. hemionus*), Père David's deer (*Elaphurus davidianus*), Reeves's muntjac (*M. reevesi*), rusa deer (*R. timorensis russa*), sambar deer (*R. unicolor*), sika deer (*C. nippon*), and white-tailed deer (*O. virginianus*), without apparent pathological consequences [21]. Genetic studies have confirmed that this sickle-cell morphology in deer represents an evolutionary adaptation involving the *β*-globin gene [38].

Automatic hematology analyzers, which estimate the cell size and volume based on electrical impedance or light scatter, are calibrated for the typical biconcave disc shape of mammalian erythrocytes [39]. Consequently, sickled or irregularly shaped cells may be incorrectly sized or counted, potentially leading to inaccurate RBC values and derived indices such as MCV, MCH, and MCHC. Likewise, alterations in white blood cell morphology—such as the presence of immature or reactive forms associated with severe or chronic inflammation—can result in misclassification or erroneous counts by automated analyzer [40–42].

## 5. Conclusion

This study establishes baseline hematological and serum biochemical profiles for the southern red muntjac (*M. muntjak*), with no significant differences observed between males and females. Variations in certain parameters compared with the northern red muntjac (*M. vaginalis*) may be attributed to differences in restraint methods and altitude. These findings provide valuable baseline data for health screening and the assessment of environmental toxicity in both captive and free-ranging individuals of this species.

## Figures and Tables

**Table 1:** Normality of the data tested by the Shapiro–Wilk test.

	Unit	*W* value	*p* value
*Hematological parameters*			
Total white blood cells	10^3^ cells/mm^3^	0.88	< 0.0001
Neutrophils	%	0.92	0.0008
Neutrophils	10^3^ cells/mm^3^	0.9	0.0001
Lymphocytes	%	0.9	0.0001
Lymphocyte	10^3^ cells/mm^3^	0.7	< 0.0001
Monocytes	%	0.91	0.0003
Monocytes	10^3^ cells/mm^3^	0.77	< 0.0001
Eosinophils	%	0.97	0.1296
Eosinophils	10^3^ cells/mm^3^	0.95	0.0129
Basophils	%	0.94	0.0073
Basophils	10^3^ cells/mm^3^	0.97	0.1246
Red blood cells	10^6^ cells/mm^3^	0.93	0.0015
Hematocrit	%	0.94	0.0041
Hemoglobin	g/dL	0.95	0.0104
Mean corpuscular volume	fL	0.98	0.6292
Mean corpuscular hemoglobin	Pg	0.99	0.9506
Mean corpuscular hemoglobin concentration	g/dL	0.99	0.9564
Red cell distribution width	%	0.98	0.3147
Platelets	×10^3^ cells/mm^3^	0.96	0.0364
Plasma protein	g/dL	0.94	0.009

*Serum biochemical parameters*			
Albumin	g/L	0.97	0.5558
Total protein	g/L	0.97	0.4883
Globulin	g/L	0.96	0.1897
Albumin/globulin ratio		0.88	0.001
Total bilirubin	μmol/L	0.91	0.0041
Alanine transaminase	U/L	0.68	< 0.0001
Alkaline phosphatase	U/L	0.78	< 0.0001
Amylase	U/L	0.44	< 0.0001
Creatinine	μmol/L	0.93	0.0225
Blood urea nitrogen	mmol/L	0.85	0.0002
BUN/CREA ratio		0.94	0.0494
Glucose	mmol/L	0.92	0.0152
Potassium	mmol/L	0.74	< 0.0001
Sodium	mmol/L	0.26	< 0.0001

*Note:p* value < 0.05 suggests a violation of the assumption of normality.

**Table 2:** Descriptive data and statistical analysis of hematology profile compared between male and female deer.

Parameters	Sex	Mean	SE	95% confidence interval		Median	SD	Min	Max	Mann–Whitney *U* test	
				Lower	Upper					*U* value	*p* value
WBC (10^3^ cells/mm^3^)	F	4.53	0.29	3.93	5.13	4.66	1.54	1.97	7.64	389.00	0.50
	M	4.56	0.39	3.77	5.36	4.22	2.16	2.07	11.24		

Neutrophils (%)	F	52.72	4.23	44.04	61.40	56.05	22.38	6.80	87.60	397.50	0.58
	M	54.85	3.90	46.88	62.83	58.40	21.73	6.80	84.80		

Neutrophils (10^3^ cells/mm^3^)	F	2.52	0.30	1.90	3.14	2.05	1.60	0.24	5.72	432.00	0.98
	M	2.68	0.38	1.91	3.46	2.36	2.11	0.18	9.52		

Lymphocytes (%)	F	37.64	3.64	30.18	45.11	33.85	19.25	9.60	86.90	419.00	0.83
	M	37.94	3.89	29.99	45.88	34.60	21.66	12.70	88.70		

Lymphocytes (10^3^ cells/mm^3^)	F	1.59	0.19	1.21	1.97	1.40	0.99	0.58	5.81	387.00	0.48
	M	1.56	0.17	1.20	1.92	1.33	0.97	0.57	4.36		

Monocytes (%)	F	3.95	0.48	2.97	4.94	3.05	2.55	0.80	9.00	382.00	0.43
	M	4.20	0.37	3.44	4.96	3.80	2.07	1.20	8.70		

Lymphocytes (10^3^ cells/mm^3^)	F	0.18	0.02	0.13	0.23	0.13	0.13	0.02	0.47	390.00	0.51
	M	0.20	0.03	0.14	0.26	0.16	0.17	0.03	0.98		

Eosinophils (%)	F	3.03	0.33	2.35	3.71	2.90	1.75	0.30	6.90	401.50	0.63
	M	2.72	0.25	2.21	3.22	2.40	1.37	0.40	5.50		

Eosinophils (10^3^ cells/mm^3^)	F	0.13	0.01	0.10	0.15	0.11	0.07	0.02	0.31	368.00	0.32
	M	0.11	0.01	0.09	0.13	0.10	0.05	0.02	0.27		

Basophils (%)	F	0.44	0.05	0.33	0.55	0.40	0.29	0.00	1.20	303.00	0.05
	M	0.30	0.04	0.21	0.38	0.30	0.24	0.00	0.90		

Basophils (10^3^ cells/mm^3^)	F	0.02	0.00	0.01	0.02	0.02	0.01	0.00	0.04	276.50	0.02
	M	0.01	0.00	0.01	0.01	0.01	0.01	0.00	0.03		

Red blood cells (10^6^ cells/mm^3^)	F	12.22	0.33	11.55	12.90	11.92	1.74	9.76	16.57	422.50	0.87
	M	12.13	0.23	11.66	12.61	11.82	1.30	10.12	16.12		

Hematocrit (%)	F	38.68	0.84	36.95	40.41	38.00	4.47	30.00	53.00	368.50	0.32
	M	39.35	0.57	38.18	40.51	39.00	3.18	33.00	50.00		

Hemoglobin (g/dL)	F	13.85	0.29	13.26	14.44	13.45	1.52	10.90	18.40	415.50	0.78
	M	13.81	0.19	13.42	14.19	13.60	1.06	12.00	15.90		

MCV (fL)	F	34.54	0.44	33.63	35.44	34.75	2.34	29.40	39.90	403.50	0.65
	M	34.83	0.44	33.94	35.72	35.00	2.43	29.30	39.60		

MCH (pg)	F	11.39	0.15	11.08	11.71	11.35	0.81	9.80	13.30	414.50	0.77
	M	11.44	0.15	11.13	11.75	11.50	0.84	9.70	13.00		

MCHC (g/dL)	F	33.00	0.13	32.73	33.26	33.10	0.69	31.50	34.20	385.00	0.46
	M	32.85	0.17	32.51	33.18	32.90	0.92	30.80	34.80		

RDW (%)	F	20.46	0.18	20.08	20.84	20.35	0.97	19.00	22.60	307.50	0.06
	M	19.97	0.18	19.60	20.34	20.00	1.01	18.20	22.20		

Platelets (10^3^ cells/mm^3^)	F	294.89	22.89	247.94	341.85	285.50	121.10	100.00	651.00	431.00	0.97
	M	287.87	17.86	251.40	324.34	291.00	99.42	74.00	554.00		

Plasma protein (g/dL)	F	6.43	0.13	6.16	6.70	6.40	0.70	5.00	7.90	374.50	0.37
	M	6.35	0.13	6.10	6.61	6.20	0.70	5.60	8.80		

*Note:p* value < 0.05 represents a significant difference (Mann–Whitney *U* test). WBC = total white blood cell count and RDW = red cell distribution width.

Abbreviations: MCH = mean corpuscular hemoglobin, MCHC = mean corpuscular hemoglobin concentration, and MCV = mean corpuscular volume.

**Table 3:** Descriptive data and statistical analysis of serum biochemistry profile compared between male (*n* = 31) and female (*n* = 28) deer.

Parameters	Sex	Mean	SE	95% confidence interval			SD	Minimum	Maximum	Mann–Whitney *U* test	
				Lower	Upper	Median				*U* value	*p* value
Albumin (g/L)	F	28.66	0.73	27.11	30.20	28.15	3.11	23.80	35.40	135.50	0.29
	M	27.42	0.46	26.45	28.39	27.70	2.01	23.10	30.30		

Total protein (g/L)	F	70.56	1.45	67.49	73.62	70.15	6.16	56.70	80.00	111.00	0.07
	M	67.83	0.82	66.10	69.55	67.00	3.58	62.00	74.70		

Globulin (g/L)	F	41.92	1.51	38.73	45.11	42.95	6.41	30.20	52.70	129.00	0.21
	M	40.41	0.57	39.21	41.60	41.10	2.48	35.40	45.00		

A/G ratio	F	0.70	0.04	0.62	0.79	0.68	0.17	0.50	1.17	165.00	0.87
	M	0.68	0.01	0.65	0.71	0.68	0.06	0.59	0.81		

Total bilirubin (μmol/L)	F	6.89	1.27	4.22	9.57	5.80	5.37	1.00	17.10	164.50	0.86
	M	7.01	1.01	4.89	9.13	6.10	4.39	1.00	14.40		

AST (U/L)	F	20.39	1.96	16.26	24.52	17.50	8.30	12.00	41.00	160.00	0.75
	M	23.05	3.52	15.66	30.44	19.00	15.33	12.00	77.00		

ALP (U/L)	F	58.78	7.76	42.42	75.14	51.50	32.90	26.00	157.00	138.50	0.33
	M	59.66	11.97	34.51	84.81	40.00	52.18	4.50	197.00		

Amylase (U/L)	F	32.83	4.21	23.94	41.72	29.50	17.88	14.00	84.00	141.50	0.38
	M	48.95	15.42	16.54	81.35	32.00	67.23	9.00	322.00		

Creatinine (μmol/L)	F	177.67	10.29	155.95	199.38	164.25	43.67	122.30	271.20	161.00	0.78
	M	164.48	7.42	148.88	180.07	173.30	32.35	96.70	250.60		

BUN (mmol/L)	F	3.41	0.55	2.25	4.57	2.75	2.34	0.95	9.15	114.00	0.09
	M	4.53	0.62	3.22	5.84	4.25	2.72	1.38	13.30		

BUN/CREA	F	19.65	3.16	12.99	26.32	15.37	13.40	5.43	58.41	106.00	0.05
	M	27.02	2.75	21.24	32.80	29.90	11.99	8.00	53.08		

Glucose (mmol/L)	F	8.22	0.52	7.13	9.31	7.80	2.19	5.80	13.77	117.00	0.10
	M	6.99	0.47	5.99	7.99	6.78	2.07	4.21	12.00		

Potassium (mmol/L)	F	3.87	0.15	3.57	4.18	3.86	0.62	2.87	4.80	129.00	0.21
	M	3.76	0.26	3.22	4.30	3.50	1.12	2.74	8.00		

Sodium (mmol/L)	F	136.61	0.44	135.67	137.54	136.50	1.88	131.10	139.70	134.00	0.27
	M	202.43	65.42	64.99	339.88	137.20	285.16	132.90	1380.00		

*Note:p* < 0.05 (Mann–Whitney *U* test) represents a significant difference. A/G = albumin/globulin ratio, ALT = alanine transaminase, ALP = alkaline phosphatase, and BUN/CREA = blood urea nitrogen/creatinine ratio.

Abbreviation: BUN = blood urea nitrogen.

**Table 4:** Hematology profile and reference intervals using 95% confidence interval, for barking deer (*n* = 59).

Parameters	Unit	Mean	SE	95% confidence interval		Median	SD	Min	Max
				Lower	Upper				
WBC	10^3^ cells/mm^3^	4.55	0.24	4.06	5.04	4.23	1.88	1.97	11.24
Neutrophils	%	53.84	2.85	48.14	59.54	56.60	21.88	6.80	87.6
Neutrophils	10^3^ cells/mm^3^	2.61	0.24	2.12	3.09	2.13	1.87	0.18	9.52
Lymphocytes	%	37.8	2.65	32.49	43.11	34.6	20.38	9.60	88.7
Lymphocytes	10^3^ cells/mm^3^	1.57	0.13	1.32	1.83	1.37	0.97	0.57	5.81
Monocytes	%	4.08	0.30	3.49	4.68	3.60	2.29	0.80	9.00
Monocytes	10^3^ cells/mm^3^	0.19	0.02	0.15	0.23	0.14	0.15	0.02	0.98
Eosinophils	%	2.87	0.20	2.46	3.27	2.60	1.56	0.3	6.90
Eosinophils	10^3^ cells/mm^3^	0.12	0.01	0.10	0.13	0.10	0.06	0.02	0.31
Basophils	%	0.36	0.04	0.29	0.43	0.30	0.27	0.00	1.20
Basophils	10^3^ cells/mm^3^	0.01	0.00	0.01	0.02	0.01	0.01	0.00	0.04
Red blood cells	10^6^ cells/mm^3^	12.17	0.20	11.78	12.57	11.86	1.51	9.76	16.57
Hematocrit	%	39.03	0.50	38.03	40.03	39.00	3.82	30.00	53.00
Hemoglobin	g/dL	13.83	0.17	13.49	14.16	13.60	1.29	10.90	18.40
MCV	fL	34.69	0.31	34.07	35.31	34.80	2.37	29.30	39.90
MCH	pg	11.42	0.11	11.20	11.63	11.40	0.82	9.70	13.30
MCHC	g/dL	32.92	0.11	32.70	33.13	33.00	0.82	30.80	34.80
RDW	%	20.2	0.13	19.94	20.47	20.10	1.01	18.20	22.60
Platelets	1000 cells/mm^3^	291.2	14.23	262.71	319.69	291.00	109.33	74.00	651.00
Plasma protein	g/dL	6.39	0.09	6.21	6.57	6.20	0.69	5.00	8.80

*Note:* WBC = total white blood cell count and RDW = red cell distribution width.

Abbreviations: MCH = mean corpuscular hemoglobin, MCHC = mean corpuscular hemoglobin concentration, and MCV = mean corpuscular volume.

**Table 5:** Serum biochemistry profile and reference intervals (95% confidence interval) for barking deer (*n* = 59).

Parameters	Unit	Mean	SE	95% confidence interval		Median	SD	Minimum	Maximum
				Lower	Upper				
Albumin	g/L	28.02	0.43	27.14	28.90	28.10	2.65	23.10	35.40
Total protein	g/L	69.15	0.84	67.45	70.86	68.80	5.12	56.70	80.00
Globulin	g/L	41.14	0.79	39.54	42.74	41.10	4.80	30.20	52.70
A/G		0.69	0.02	0.65	0.73	0.68	0.12	0.50	1.17
TB (μmol/L)	μmol/L	6.95	0.79	5.35	8.56	6.10	4.82	1.00	17.10
AST	U/L	21.76	2.03	17.65	25.87	19.00	12.33	12.00	77.00
ALP	U/L	59.23	7.11	44.80	73.66	43.00	43.28	4.50	197.00
Amylase	U/L	41.11	8.18	24.51	57.70	32.00	49.78	9.00	322.00
Creatinine	μmol/L	170.89	6.30	158.12	183.67	171.20	38.32	96.70	271.20
BUN	mmol/L	3.99	0.42	3.13	4.84	3.34	2.57	0.95	13.30
BUN/CREA		23.44	2.15	19.08	27.79	21.56	13.06	5.43	58.41
Glucose	mmol/L	7.59	0.36	6.86	8.32	7.47	2.19	4.21	13.77
Potassium	mmol/L	3.82	0.15	3.52	4.12	3.66	0.90	2.74	8.00
Sodium	mmol/L	170.41	33.60	102.26	238.55	136.80	204.39	131.10	1380.00

*Note:* A/G = albumin/globulin ratio, ALT = alanine transaminase, ALP = alkaline phosphatase, and BUN/CREA = blood urea nitrogen/creatinine ratio.

Abbreviation: BUN = blood urea nitrogen.

**Table 6:** Comparison between the hematological parameters of *M. muntjak* and other related species.

**Countries**	**Thailand**										**India**	

Authors	This study		[19]		[17]		[18]		[20]		[16]	
Number of animals	59		10		114		20		6		4	
Restraint	Chemical		Physical		Physical		Chemical		Physical		Physical	
Species	*Muntiacus muntjak*		*Rusa unicolor equinus*		*Rusa timorensis russa*		*Rucervus eldii thamin*		*Capra hircus*		*Muntiacus vaginalis*	

**Parameters**	**Mean**	**SD**	**Mean**	**SD**	**Mean**	**SD**	**Mean**	**SD**	**Mean**	**SD**	**Mean**	**SD**

WBC (10^3^ cell/mm^3^)	4.55	1.88	10.03	NP	5.94	NP	NP	NP	6.14	1.37	2.64	0.35
NEU (10^3^ cell/mm^3^)	2.61	1.87	4.66	1.54	2.44	NP	NP	NP	2.82	0.63	1.40	0.19
LYM (10^3^ cell/mm^3^)	1.57	0.97	4.89	1.55	3.27	NP	NP	NP	3.01	0.67	1.08	0.10
MON (10^3^ cell/mm^3^)	0.19	0.15	0.20	1.63	0.08	NP	NP	NP	0.12	0.03	0.11	0.05
EOS (10^3^ cell/mm^3^)	0.12	0.06	0.21	2.18	0.18	NP	NP	NP	0.12	0.03	0.03	0.03
BAS (10^3^ cell/mm^3^)	0.01	0.01	0.04	0.51	0.07	NP	NP	NP	NC	NC	0.00	0.01
RBC (10^6^ cell/mm^3^)	12.17	1.51	8.29	1.68	11.49	NP	NP	NP	3.26	0.8	21.34	0.72
HCT (%)	39.03	3.82	67.99	16.42	40.55	NP	39.74	5.65	25.67	0.58	52.38	0.75
HB (g/dL)	13.83	1.29	12.50	2.87	13.25	NP	12.31	1.80	9.70	0.40	15.79	0.36
MCV (fL)	34.69	2.37	84.27	21.28	40.80	NP	NP	NP	81.43	86.23	24.63	0.91
MCH (pg)	11.42	0.82	15.23	2.47	11.55	NP	NP	NP	30.67	5.86	7.43	0.33
MCHC (g/dL)	32.92	0.82	18.76	3.70	28.50	NP	NP	NP	37.77	0.90	30.14	0.29
RDW (%)	20.20	1.01	28.24	3.69	19.90	NP	NP	NP	NP	NP	NP	NP
PLT (10^3^ cell/mm^3^)	291.20	109.33	279.9	143.18	230.50	NP	NP	NP	NP	NP	NP	NP
Plasma protein (g/dL)	6.39	0.69	3.60	3.60	NP	NP	NP	NP	NP	NP	NP	NP

*Note:* WBC = total white blood cell count, NEU = neutrophil count, LYM = lymphocyte count, EOS = eosinophil count, MON = monocyte count, basophil count, HCT = hematocrit, RBC = red blood cell count, HB = hemoglobin, RDW = red cell distribution width, PLT = platelet count, and NP = no provided data.

Abbreviations: MCH = mean cell hemoglobin, MCHC = mean cell hemoglobin concentration, and MCV = mean cell volume.

**Table 7:** Comparison between the serum biochemistry of *M. muntjak* and other species.

**Countries**	**Thailand**								**India**	

Authors	This study		[19]		[18]		[20]		[16]	
Number of animals	59		10		20		6		4	
Restraint	Chemical		Physical		Chemical		Physical		Physical	
Species	*Muntiacus muntjak*		*Rusa unicolor equinus*		*Rucervus eldii thamin*		*Capra hircus*		*Muntiacus vaginalis*	

**Parameters**	**Mean**	**SD**	**Mean**	**SD**	**Mean**	**SD**	**Mean**	**SD**	**Mean**	**SD**

TP (g/L)	69.15	5.12	60.90	13.60	61.31	5.84	65.90	4.40	65.10	2.40
ALB (g/L)	28.02	2.65	25.40	7.90	NP	NP	37.80	1.00	38.70	1.90
GLOB(g/L)	41.14	4.80	36.00	6.99	NP	NP	28.10	3.30	26.50	2.20
A/G ratio	0.69	0.12	0.71	1.13	NP	NP	1.35	0.30	1.40	0.21
TB (μmol/L)	6.95	4.82	10.26	11.12	NP	NP	NP	NP	8.04	2.06
ALT (U/L)	NP	NP	43.00	11.67	23.95	7.49	20.33	1.53	42.5	1.71
ALP (U/L)	59.23	43.28	196.50	174.22	71.02	41.96	NP	NP	NP	NP
AST (U/L)	21.76	12.33	69.70	24.57	62.85	20.60	NP	NP	58	1.15
AMY (U/L)	41.11	49.78	NP	NP	NP	NC	NP	NP	NP	NP
CREA (μmol/L)	170.89	38.32	75.10	11.50	227.48	26.48	NP	NP	157.31	10.61
BUN (mmol/L)	3.99	2.57	6.43	2.13	5.92	1.30	7.11	1.64	10.55	0.57
BUN/CREA	23.44	13.06	8.56	18.52	2.60	4.91	NP	NP	6.71	5.37
GLU (mmol/L)	7.59	2.19	2.85	1.51	6.62	1.93	NP	NP	5.16	0.36
K+ (mmol/L)	3.82	0.90	NP	NP	4.20	1.17	NP	NP	NP	NP
Na+ (mmol/L)	170.41	204.39	NP	NP	111.56	14.97	NP	NP	NP	NP
Ca+ (mmol/L)	NP	NP	1.34	0.51	1.86	0.27	NP	NP	2.48	0.02

*Note:* NP = not provided data, ALB = albumin, GLO = globulin, albumin/globulin ratio (A/G), ALP = alkaline phosphatase, ALT = alanine transaminase, CREA = creatinine, GLU = glucose, AMY = amylase (AMY), Na^+^ = sodium, and K^+^ = potassium.

Abbreviations: BUN = blood urea nitrogen, TB = total bilirubin, and TP = total protein.

## Data Availability

The data that support the findings of this study are available from the corresponding author upon reasonable request.
